# The incidence and prevalence of hospital-acquired (carbapenem-resistant) *Acinetobacter baumannii* in Europe, Eastern Mediterranean and Africa: a systematic review and meta-analysis

**DOI:** 10.1080/22221751.2019.1698273

**Published:** 2019-12-05

**Authors:** Olaniyi Ayobami, Niklas Willrich, Thomas Harder, Iruka N. Okeke, Tim Eckmanns, Robby Markwart

**Affiliations:** a Unit 37: Nosocomial Infections, Surveillance of Antimicrobial Resistance and Consumption, Robert Koch-Institute, Berlin, Germany; b Department of Pharmaceutical Microbiology, Faculty of Pharmacy, University of Ibadan, Ibadan, Nigeria

**Keywords:** *Acinetobacter baumannii*, hospital-acquired infections, healthcare-acquired infections, nosocomial infections, carbapenem resistance

## Abstract

Due to therapeutic challenges, hospital-acquired infections (HAIs) caused by *Acinetobacter baumannii* (HA-AB), particularly carbapenem-resistant strains (HA-CRAB) pose a serious health threat to patients worldwide. This systematic review sought to summarize recent data on the incidence and prevalence of HA-AB and HA-CRAB infections in the WHO-defined regions of Europe (EUR), Eastern Mediterranean (EMR) and Africa (AFR). A comprehensive literature search was performed using MEDLINE, EMBASE and GMI databases (01/2014-02/2019). Random-effects meta-analyses were performed to determine the pooled incidence of HA-AB and HA-CRAB infections as well as the proportions of *A. baumannii* among all HAIs. 24 studies from 3,340 records were included in this review (EUR: 16, EMR: 6, AFR: 2). The pooled estimates of incidence and incidence density of HA-AB infection in intensive care units (ICUs) were 56.5 (95% CI 33.9-92.8) cases per 1,000 patients and 4.4 (95% CI 2.9-6.6) cases per 1,000 patient days, respectively. Five studies conducted at a hospital-wide level or in specialized clinical departments/wards (ICU + non-ICU patients) showed HA-AB incidences between 0.85 and 5.6 cases per 1,000 patients. For carbapenem-resistant *A. baumannii* infections in ICUs, the pooled incidence and incidence density were 41.7 (95% CI 21.6-78.7) cases per 1,000 patients and 2.1 (95% CI 1.2-3.7) cases per 1,000 patient days, respectively. In ICUs, *A. baumannii* and carbapenem-resistant *A. baumannii* strains accounted for 20.9% (95% CI 16.5-26.2%) and 13.6% (95% CI 9.7-18.7%) of all HAIs, respectively. Our study highlights the persistent clinical significance of hospital-acquired *A. baumannii* infections in the studied WHO regions, particularly in ICUs.

## Introduction

*Acinetobacter* spp. are non-fermenting, largely opportunistic Gram-negative bacteria that are ubiquitous in the environment. *Acinetobacter baumannii* complex (*Acinetobacter nosocomialis, Acinetobacter pitti* and *Acinetobacter baumannii*) are the most clinically significant out of the over 50 species in the *Acinetobacter* genus [1]. Of all *Acinetobacter* species, *A. baumannii* sensu-stricto (shortened to *A. baumannii*) is responsible for about 90% of the clinical infections caused by *Acinetobacter* spp. in humans [1]. Infections from *A. baumannii*, in particular carbapenem-resistant *A. baumannii* (CRAB), are of significant public health importance worldwide because of their association with high treatment costs, mortality and morbidity [2,3]. The World Health Organization (WHO) has ranked carbapenem-resistant *A. baumannii* as a critical priority pathogen on its global priority list of antibiotic-resistant bacteria to guide drug research and development [4]. Worldwide, CRAB has been implicated in several hospital outbreaks of diseases like pneumonia, bloodstream, wound and urinary tract infections, especially among patients with severe morbidities such as those staying in ICU [5,6]. The pathogen is able to survive adverse environmental conditions, fostering its persistence and spread in the hospital environment [7,8]. Treating carbapenem-resistant *A. baumannii* infections is very challenging since they are naturally resistant to antibiotics in the WHO “Access” and “Watch” list. They are therefore associated with poor clinical outcomes across many healthcare settings [9]. Multiple studies have been conducted worldwide to analyses the burden of healthcare-associated infections caused by *A. baumannii*. Depending on the variables under study (e.g. study location, included patient cohorts and clinical wards), the incidence or prevalence estimates of (carbapenem-resistant) *A. baumannii* vary significantly between the different studies. Even though representative data are scarce, there is evidence for increasing incidence of *A. baumannii* outbreaks in healthcare facilities across Sub-Saharan Africa and Eastern Mediterranean countries, both of which have diverse populations [10,11]. Moreover, the epidemiological situation has worsened in Europe as it has become endemic in the south and eastern parts of Europe with some areas experiencing cross-border transmission [1].

To our knowledge, no systematic review on the frequency of (carbapenem-resistant) *A. baumannii* across Europe, the Eastern Mediterranean Region and Africa has been published. A comprehensive understanding of the current epidemiological picture is therefore needed. This systematic review aims to comprehensively analyse recent data on the prevalence and incidence of hospital-acquired infections with (carbapenem-resistant) *A. baumannii* to better understand the current epidemiology in these WHO-defined regions. This will help with the development of context-specific prevention and control interventions against this dangerous and increasingly untreatable pathogen.

## Materials and methods

This systematic review was registered in the Prospective Register of Systematic Reviews (PROSPERO; registration no. CRD42019124503) and conducted using the PRISMA checklist [12].

### Search strategy

In order to identify recent studies on the burden of hospital-acquired *A. baumannii* infections, we performed a systematic literature search using the electronic databases MEDLINE, EMBASE, and The Global Index Medicus (GIM) for studies from January 2014 up to February 2019. For the search strings, we used a combination of synonyms of hospital-acquired infections and *Acinetobacter baumannii* (see additional file 1). Title and abstract screening and full text review of potentially eligible studies were carried out independently by two researchers (R. M. and O. A.). All discrepancies regarding the inclusion were resolved through discussion.

### Inclusion / exclusion criteria

We included epidemiological studies that provide data on the prevalence and/or incidence of HA-AB and HA-CRAB infections, regardless of whether the studies were retrospective or prospective. Studies were eligible if they reported relevant data from patient cohorts from entire hospitals or individual clinical departments/wards without selection of particular diseases or patient sub-groups. Studies were only included if they were conducted in the following WHO regions: African Region (AFR), Eastern Mediterranean Region (EMR) and European Region (EUR). A study was eligible if it provided a clear distinction between *A. baumannii* infections acquired in the hospital and community. Only studies written in English, German and French were eligible. Since this systematic review focused on recent epidemiological *A. baumannii* data, studies published before 2014 and studies that completed data collection before 2009 were excluded. Studies with any of the following study design/study type were excluded: Reviews, systematic reviews, editorials, letters, commentaries, conference abstracts, case reports, case–control studies and randomized controlled trials (RCTs). Studies were excluded if no clear distinction was made between *A. baumannii* infection and colonization status.

### Data extraction and outcomes of interest

Two authors (R. M. and O. A.) independently extracted data using a standard extraction form. Any discrepancies were resolved through discussion. The primary outcome was the prevalence and/or incidence of hospital-acquired (carbapenem-resistant) *A. baumannii* infections among (i) all patients in the hospital or individual departments/wards and (ii) among all hospital-acquired infections (HAIs). The prevalence refers to the number of HA-(CR)AB cases per 1,000 patients present in the hospital or ward at a given time point. The incidence refers to the number of new cases acquiring a HA-(CR)AB infection per 1,000 patients followed-up for a defined period of time. Other primary outcome parameters of interest were incidence density and population-based estimates of HA-AB and HA-CRAB infections. Secondary outcomes were attributable mortality and/or lethality as well as morbidity of HA-AB and HA-CRAB infections. A case of *A. baumannii* infection was categorized as “carbapenem-resistant” when stated so by the authors of the study or when carbapenem resistance data of *A. baumannii* isolates were provided in the studies. If antimicrobial susceptibility testing showed resistance for at least one carbapenem (such as meropenem, imipenem), the isolate was categorized as carbapenem-resistant. In addition to the *a priori* analysis plan outlined in the study protocol, we also performed subgroup analyses, stratifying countries according to income level as defined by the World Bank (https://datatopics.worldbank.org/world-development-indicators/stories/the-classification-of-countries-by-income.html).

### Risk of bias assessment and statistical analysis

All included studies were assessed for risk of bias by two review authors (R.M. and O.A.). Disagreements were resolved through discussion. The risk of bias tool developed by Hoy et al. was used, which is specific for the study designs of the included studies [13]. In order to calculate pooled incidences and incidence densities, raw proportions were logit transformed [14]. For all analyses, random-effects models with inverse variance weighting were used. The I^2^ statistic was used to estimate inter-study heterogeneity. All statistical analyses and forest plots visualizations were performed using R version 3.5.1 [15] with the “meta” (v. 4.9.5) and “metafor” (v. 2.1.0) packages.

## Results

**Figure 1.**
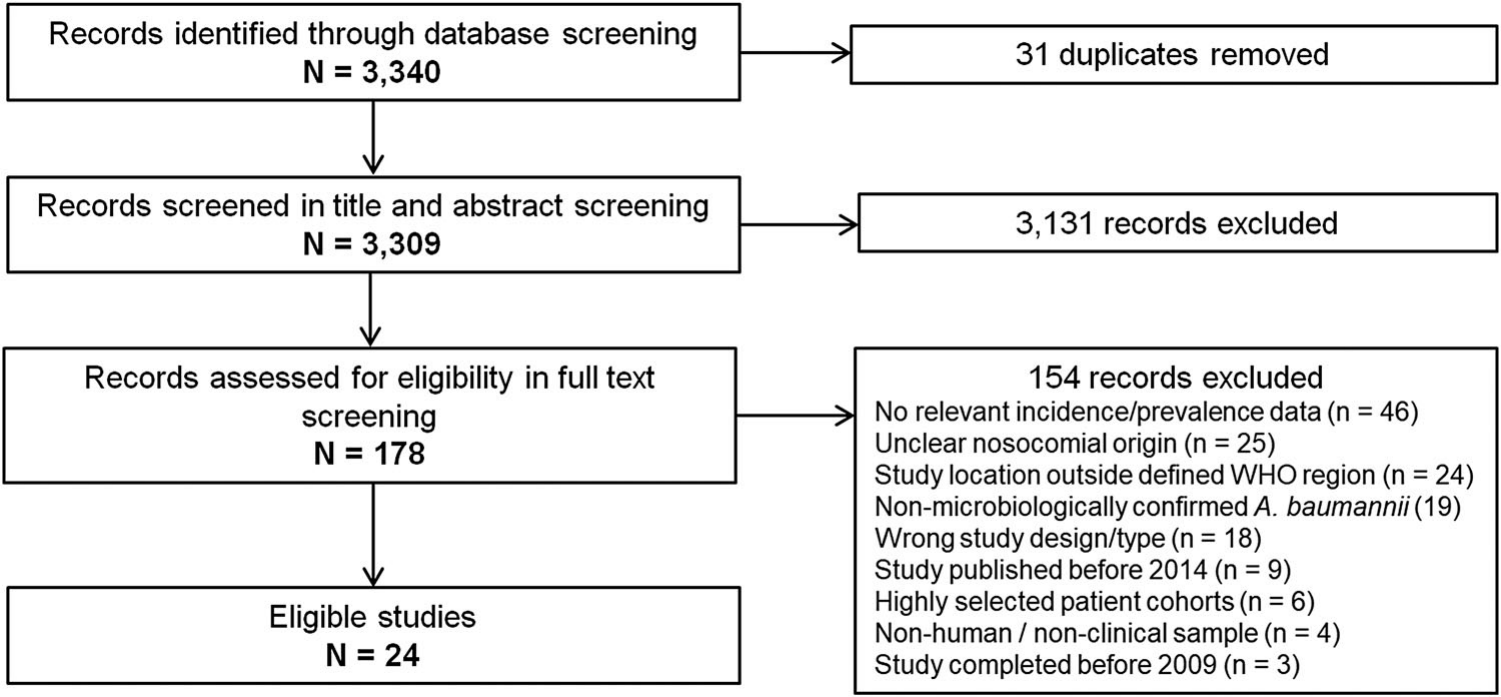
Study selection flowchart.

In total, 3,340 records were identified through our database search. Following screening of titles and abstracts, 178 potentially eligible studies were identified and included for full text review. Finally, 24 studies published between 2014 and 2018 were found eligible and therefore included in this systematic review (Figure 1).

### Study characteristics and risk of bias assessment

The study characteristics are summarized in Table 1. Among the 24 included studies, 16 were conducted in the European region of the WHO. Of these, eight studies were carried out in Turkey. Six out of 24 studies were conducted in the Eastern Mediterranean region and two in the African region. The majority of studies were from upper-middle income (*n* = 11) and high income (*n* = 8) countries. Two studies reported prevalence data and 22 studies provided data on the incidence and/or incidence density of hospital-acquired *A. baumannii* infections. Just over half of the included studies (*n* = 13) were conducted exclusively on intensive care units (ICU). Five studies reported hospital-level data, while six studies were carried out in other wards or specialized clinic departments where both ICU and non-ICU patients are seen. The majority of studies (*n* = 16) also reported data on hospital-acquired infections with carbapenem-resistant *A. baumannii*. None of the included studies provided data on population-based incidence/prevalence or attributable mortality and morbidity.

**Table 1.** Study characteristics of the included studies.

Study	Country (WHO region, income level)	Study design	Study time (study duration)	Description of medical wards / patients	HAI definition	Sample size
*ICU-based studies*						
Atici [16]	Italy (EUR, high income)	Incidence study; multicenter	2006–2013 (4 × 6 months)	Patients from 75 ICUs from 52 hospitals (SPIN-UTI project)	ECDC protocol [17]	10,703 patients; 2,255 HAIs
Atici [18]	Turkey (EUR, upper-middle income)	Incidence study; single centre	2011–2014 (47 months)	Children from a 14-bed pediatric ICU in a university hospital	CDC/NHSN criteria [19]	1,007 patients; 224 HAIs
Custovic [20]	Bosnia and Herzegovina (EUR, upper-middle income)	Incidence study; single centre	2013 (12 months)	Patients from the Clinic of Anesthesiology and Reanimation in a university hospital	CDC/NHSN criteria	855 patients; 105 patients with HAIs
Duszynska [21]	Poland (EUR, high income)	Incidence study; single centre	2011–2016 (72 months)	Patients from a 20-bed ICU in a university hospital	CDC/NHSN criteria [22]	2,459 patients; 589 HAIs
El-Nawawy [23]	Egypt (EMR, lower-middle income)	Incidence study; single centre	2016 (12 months)	Children (0-15yrs) from a 9-bed pediatric ICU in a university hospital	CDC/NHSN criteria [24]	264 patients; 36 HAIs
Kolpa [25]	Poland (EUR, high income)	Incidence study; single centre	2007–2016 (120 months)	Adult patients from a 9-bed ICU in a non-teaching secondary hospital	ECDC protocol [26]	1,847 patients; 510 HAIs
Kostakoglu [27]	Turkey (EUR, upper-middle income)	Incidence study; single centre	2013 (12 months)	Patients from 4 ICUs (46 adult beds) in a training and research hospital	CDC/NHSN criteria [22]	566 patients; 309 HAIs
Nageeb [28]	Egypt (EMR, lower-middle income)	Incidence study; single centre	2011–2012 (9 months)	Random samples of patients with nosocomial infections from different ICUs in a university hospital	Infection occurring 48hrs after admission	350 HAIs
Öncül [29]	Turkey (EUR, upper-middle income)	Incidence study; single centre	2001–2012 (108 months)	Burn patients from a 9-bed burn ICU in a military academy hospital	CDC/NHSN criteria [30]	658 patients; 602 HAIs
Sileem [31]	Saudi Arabia (EMR, high income)	Incidence study; single centre	2014–2015 (12 months)	Patients from ICUs in a general hospital	As defined by Vincent et al. [32]	650 patients; 78 patients with HAIs
Uwingabiye [33]	Morocco (EMR, lower-middle income)	Incidence study; single centre	2015–2016 (19 months)	Patients from two 10-bed ICUs (medical and surgical) in a military teaching hospital	CDC/NHSN criteria [22]	964 patients
Yetkin [34]	Turkey (EUR, upper-middle income)	Incidence study; single centre	2007–2015 (96 months)	Patients from all ICUs in a regional referral tertiary hospital	CDC/NHSN criteria [19,22]	48,263 patients; 4,272 HAIs
Yilmaz [35]	Turkey (EUR, upper-middle income)	Incidence study; single centre	2010 (6 months)	Adult patients from a general medicine ICU in a university hospital	Infections occurring 48hrs after admission	269 patients; 109 HAI patients
*Hospital-based studies*						
Ahoyo [36]	Benin (AFR, low income)	Prevalence study; multicenter	2012 (1 d)	Patients from 39/45 hospitals in Benin	CDC/NHSN criteria [30]	3,130 patients; 972 HAIs
Gashaw [37]	Ethiopia (AFR, low income)	Incidence study; single centre	2016 (5 months)	Patients from all wards in a university hospital	Not reported	1,105 patients; 126 HAIs
Kolpa [38]	Poland (EUR, high income)	Incidence study; single centre	2012–2016 (48 months)	Non-teaching secondary care hospital	ECDC protocol [39]	159,028 patients; 2,184 HAIs
Kritsotakis [40]	Greece (EUR, high income)	Prevalence study; multicenter	2012 (1 d)	A nationally representative cross-section of all patients hospitalized in 37 acute care hospitals	ECDC protocol [41]	8,247 patients; 820 HAIs
Matta [42]	Lebanon (EMR, upper-middle income)	Incidence study; multicenter	Not reported (6 months)	Adult patients with infections from 3 private university hospitals and 2 private non-university hospitals	CDC/NHSN criteria [22]	116 patients with HAIs
*Studies conducted in specialized clinical departments/wards with ICU- and non-ICU-patients*						
Armin [43]	Iran (EMR, upper-middle income)	Incidence study; multicenter	2014–2015 (6 months)	Patients with nosocomial infections admitted to surgery, neurosurgery, internal medicine, ICU and post coronary care unit wards in 3 hospitals	Culture-proven infections occurring 48hrs after admission	530 HAIs
Atilla [44]	Turkey (EUR, upper-middle income)	Incidence study; single centre	2009–2011 (29 months)	Patients from a burn unit with an ICU with 4 beds and 9 single rooms in an education and training hospital	CDC/NHSN criteria [22]	465 patients; 68 HAIs
Gecgel [45]	Turkey (EUR, upper-middle income)	Incidence study; single centre	2011–2015 (58 months)	Patients from clinical departments and Cardiology and Cardiovascular surgery ICUs in a training and research hospital	CDC/NHSN criteria	27,886 patients; 606 HAIs
Kuzdan [46]	Turkey (EUR. Upper-middle income)	Incidence study; single centre	2008–2010 (26 months)	Children from pediatric units consisting of 28 beds and 6 rooms and one pediatric intensive care unit of an university hospital	CDC/NHSN criteria [19]	2,350 patients; 389 HAIs
Walaszek [47]	Poland (EUR, high income)	Incidence study; single centre	2003–2012	Patients from the 46-neurosurgery ward (including 6-bed ICU) in a district hospital	CDC/NHSN criteria and ECDC protocol [41]	13,3551 patients; 516 HAIs
*Studies conducted in specialized wards with non-ICU-patients only*						
Cei [48]	Italy (EUR, high income)	Incidence study; single centre	2009–2011 (23 months)	Patients with consecutive recorded bacterial and fungal isolates from an internal medicine ward	CDC/NHSN criteria [22]	249 HAIs

Overall, risk of bias was low, as judged by the risk of bias tool by Hoy et al. (additional File 2, additional Fig.1) [13]. This is partly explained because, as per protocol, only epidemiological studies that included largely unselected patient cohorts from whole hospitals or individual departments/wards were eligible. The majority of studies (19 out of 24) used criteria from the Centers for Disease Control and Prevention / National Healthcare Safety Network (CDC/NHSN) or European Centre for Disease Prevention and Control (ECDC) to define hospital-acquired infections. The CDC/NHSN and ECDC definitions of HAIs are widely used and validated tools in the surveillance of healthcare-acquired infections. However, except for three multicenter studies that provided nationally representative estimates, the country representativeness was unclear or was not met in the majority of the included studies.

### Incidence of hospital-acquired *Acinetobacter baumannii* infections

**Figure 2.**
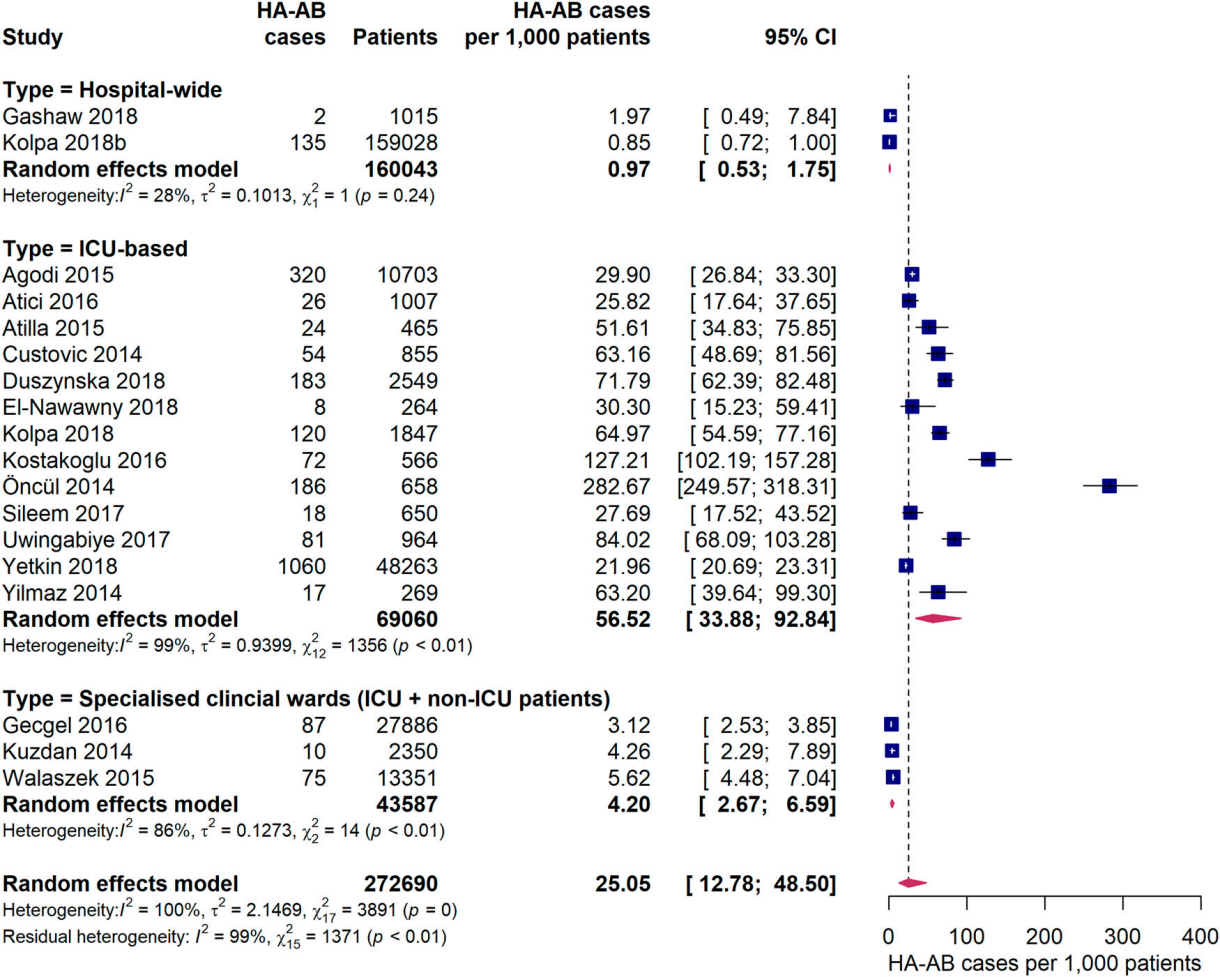
Forest plot of studies on the incidence of hospital-acquired *Acinetobacter baumannii* infections. Abbreviations: HA-AB: hospital-acquired *Acinetobacter baumannii*, ICU: intensive care unit, 95% CI: 95% confidence interval.

Based on data from 18 studies, the overall pooled incidence of hospital-acquired *A. baumannii* infections (HA-AB) in the WHO regions of Europe, Eastern Mediterranean and Africa is 25.1 (95% CI 12.8-48.5) cases per 1,000 patients (Figure 2). However, the *I*^2^ statistics indicates considerable statistical heterogeneity between the studies. In particular, incidence estimates differed substantially between studies that were conducted exclusively in intensive care units and studies that additionally included data from non-intensive care units. The pooled incidence of HA-AB infections in ICUs is 56.5 (95% CI 33.9-92.8) cases per 1,000 ICU patients. It is important to note that the between-study variation (*I*^2^ = 99%, *p* < 0.01) was also substantial within the ICU-based study set. Further analyses revealed no statistically significant differences in ICU-based rates of HA-AB infections between the EUR and EMR WHO regions (61.4 [95% CI 33.9-100.9] vs. 42.8 [95% CI 18.0-98.4], *Q* = 0.47, *p* = 0.495). In addition, no differences in the incidence of ICU HA-AB infections were observed across country income levels (high income [four studies], upper-middle income [seven studies] and lower-middle income countries [two studies], *Q* = 0.43, *p* = 0.8076).

Compared to ICU-based estimates, incidences for HA-AB infections were up to 10 times smaller in three studies conducted in specialist clinical departments/wards where both ICU and non-ICU patients were seen. In these studies, HA-AB incidences ranged between 3.1 and 5.6 per 1,000 patients (pooled estimate: 4.2 [95% CI 2.7-6.6] per 1,000 patients). With a pooled incidence of 0.97 (95% CI 0.53-1.8) per 1,000 patients, the lowest HA-AB infection incidences were observed in hospital-wide studies.

**Figure 3.**
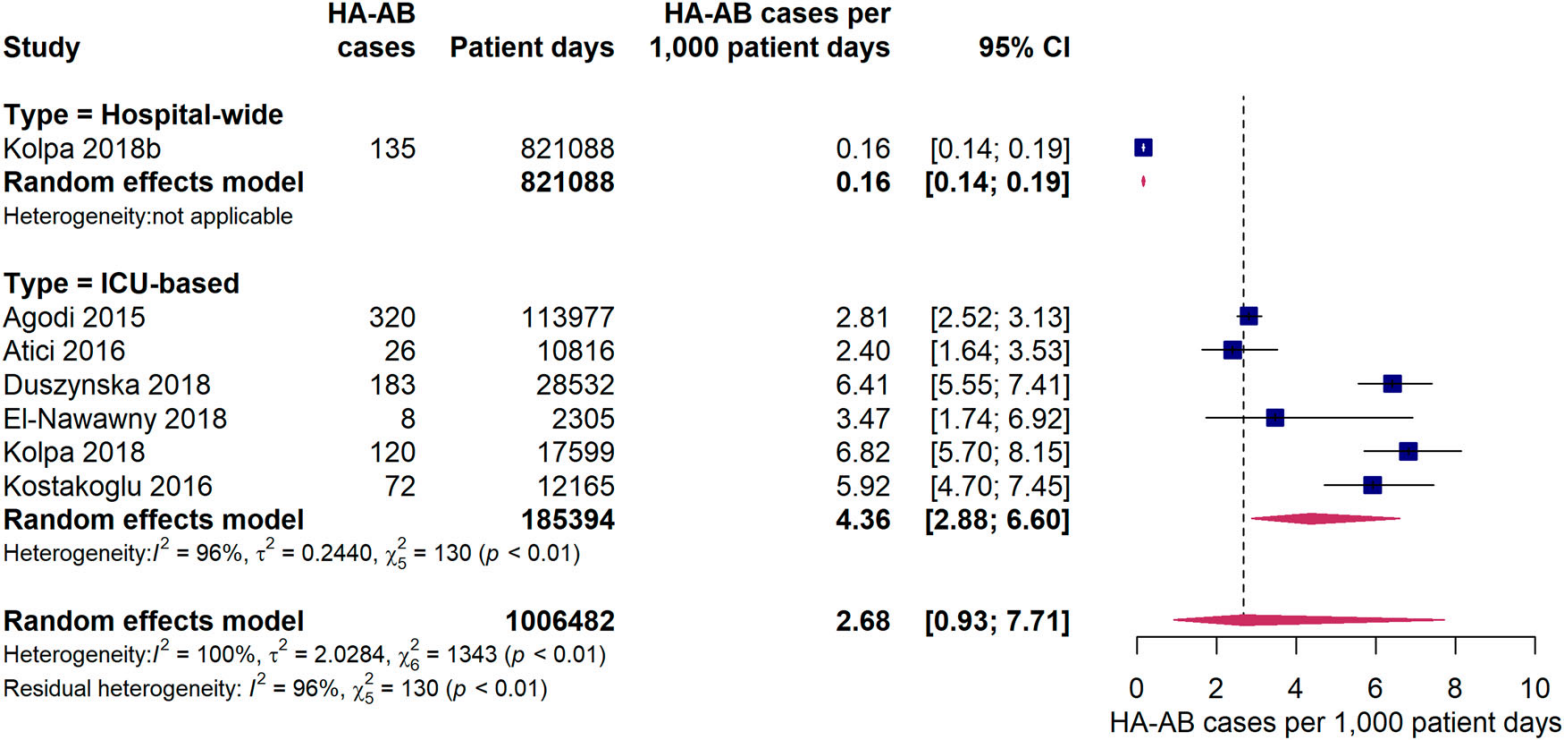
Forest plot of studies on the incidence density of hospital-acquired *Acinetobacter baumannii* infections. Abbreviations: HA-AB: hospital-acquired *Acinetobacter baumannii*, ICU: intensive care unit, 95% CI: 95% confidence interval

Six studies reported data on the incidence density of hospital-acquired *A. baumannii* infections on ICUs expressed as HA-AB cases per 1,000 patient days. The pooled estimate was 4.4 (95% CI 2.9-6.6) HA-AB cases per 1,000 ICU patient days (Figure 3). The individual study estimates ranged from 2.8 to 6.8 HA-AB cases per 1,000 ICU patient days, with a considerably high inter-study heterogeneity (*I*^2^ = 96%, *p* < 0.01). One included study reported a hospital-wide incidence density of 0.16 (95% CI 0.14-0.19) HA-AB infections per 1,000 patient days, which was markedly lower than the ICU estimates.

### Incidence of hospital-acquired carbapenem-resistant *Acinetobacter baumannii* infections

**Figure 4.**
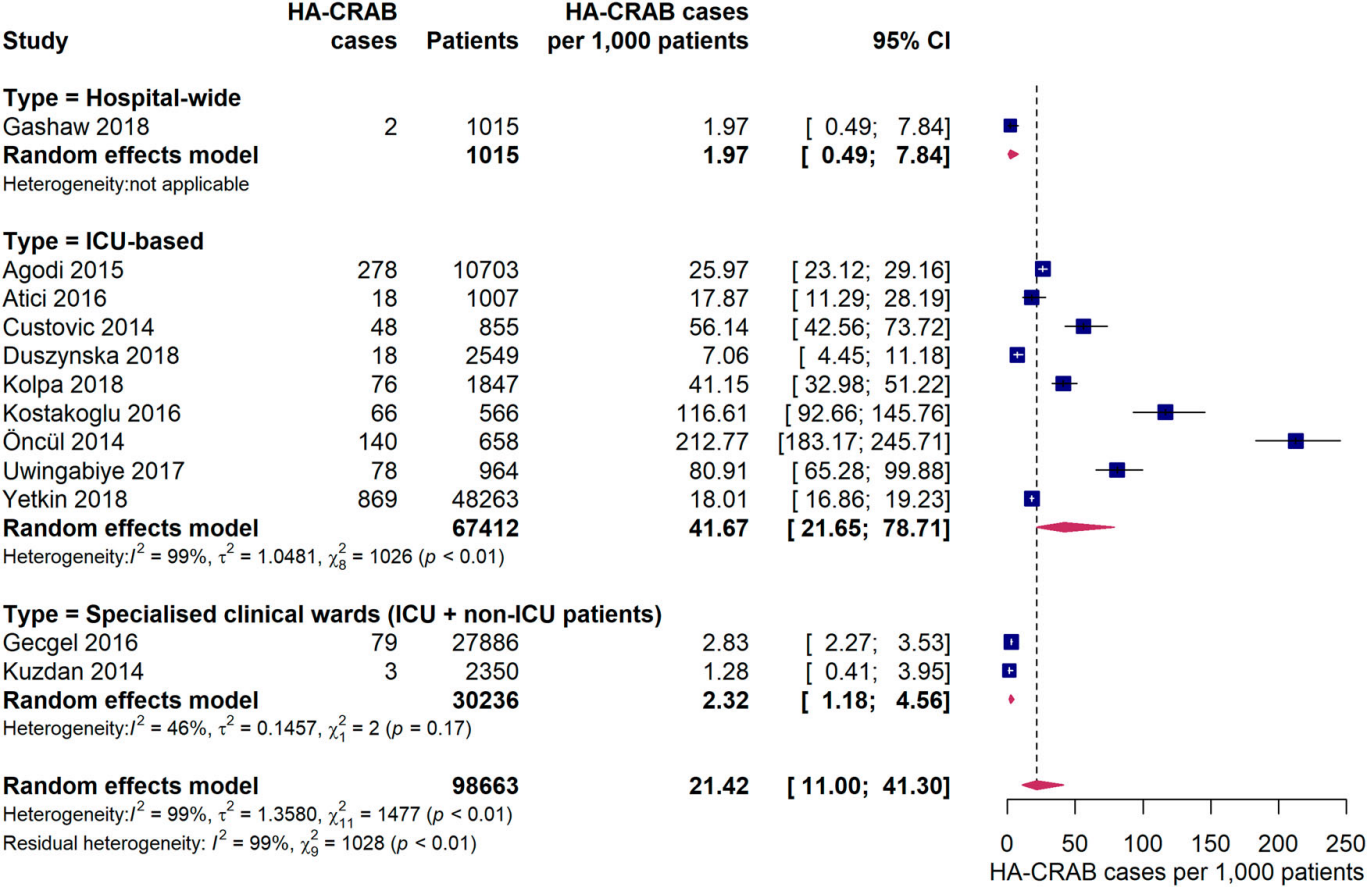
Forest plot of studies on the incidence of hospital-acquired carbapenem-resistant *Acinetobacter baumannii* infections. Abbreviations: HA-CRAB: hospital-acquired carbapenem-resistant *Acinetobacter baumannii*, ICU: intensive care unit, 95% CI: 95% confidence interval.

Twelve studies provided data on the incidence of hospital-acquired infections with carbapenem-resistant *A. baumannii* (HA-CRAB). The pooled incidence of HA-CRAB cases was 21.4 (95% CI 11.0-41.3) cases per 1,000 patients in the EUR, EMR and AFR WHO regions (Figure 4). Estimates of HA-CRAB incidences were substantially higher in studies exclusively conducted on ICUs compared to hospital-wide studies or studies carried out in specialist clinical departments with ICU and non-ICU patients. The pooled incidence of HA-CRAB infections in ICUs was 41.7 (95% CI 21.7-78.7) cases per 1,000 ICU patients, although a considerably high inter-study variation was observed (*I*^2^ = 99%, *p* < 0.01). Much lower HA-CRAB infection rates were observed in one hospital-wide study (2.0 [95% CI 0.49-7.8] cases per 1,000 admissions) and in two studies conducted in specialist clinical departments for ICU and non-ICU patients (pooled estimate: 2.3 [95% CI 1.2-4.6] cases per 1,000 patients). Since eight out of nine studies were conducted in Europe, it was not possible to meaningfully compare ICU-based HA-CRAB incidences between the WHO regions. However, we found that ICU HA-CRAB incidences tended to be higher in upper-middle income countries (five studies, 55.8 cases per 1,000 ICU patients [95% CI 15.6-180.3]) than in high income countries (three studies, 20.6 cases per 1,000 ICU admissions [95% CI 10.7-39.0]), although the difference was not statistically significant (*Q* = 1.90, *p* = 0.1681). When compared to the pooled ICU HA-CRAB incidence from high income countries, the one study from a lower-middle income country (Morocco) [33] was markedly higher (80.9 cases per 1,000 ICU patients [95% CI 65.3-99.9]).

**Figure 5.**
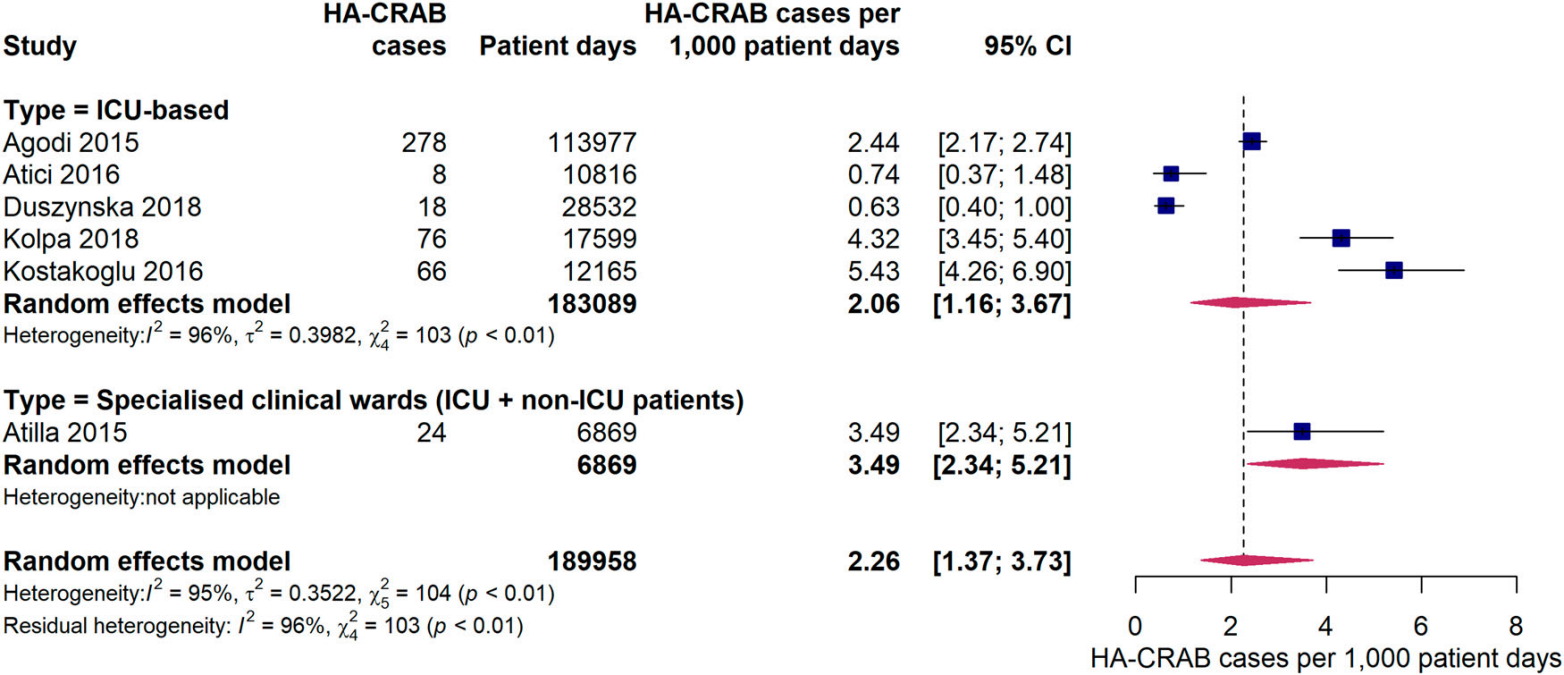
Forest plot of studies on the incidence density of hospital-acquired carbapenem-resistant *Acinetobacter baumannii* infections. Abbreviations: HA-CRAB: hospital-acquired carbapenem-resistant *Acinetobacter baumannii*, ICU: intensive care unit, 95% CI: 95% confidence interval.

Five studies provided data on the incidence density of hospital-acquired carbapenem-resistant *A. baumannii* infections on ICUs. The pooled estimate was 2.1 (95% CI 1.2-3.7) HA-CRAB cases per 1,000 ICU patient days (Figure 5), with individual study estimates ranging between 0.63 and 5.4 cases per 1,000 patient ICU days. A considerable inter-study variation was observed (*I*^2^ = 96%, *p* < 0.01). The study by Atilla et al. 2015 [44] which was conducted on a specialist burn unit with both ICU and non-ICU patients reported an incidence density of HA-CRAB infections of 3.5 per 1,000 patient days.

### Proportions of hospital-acquired *Acinetobacter baumannii* infections among all healthcare-acquired infections

**Figure 6.**
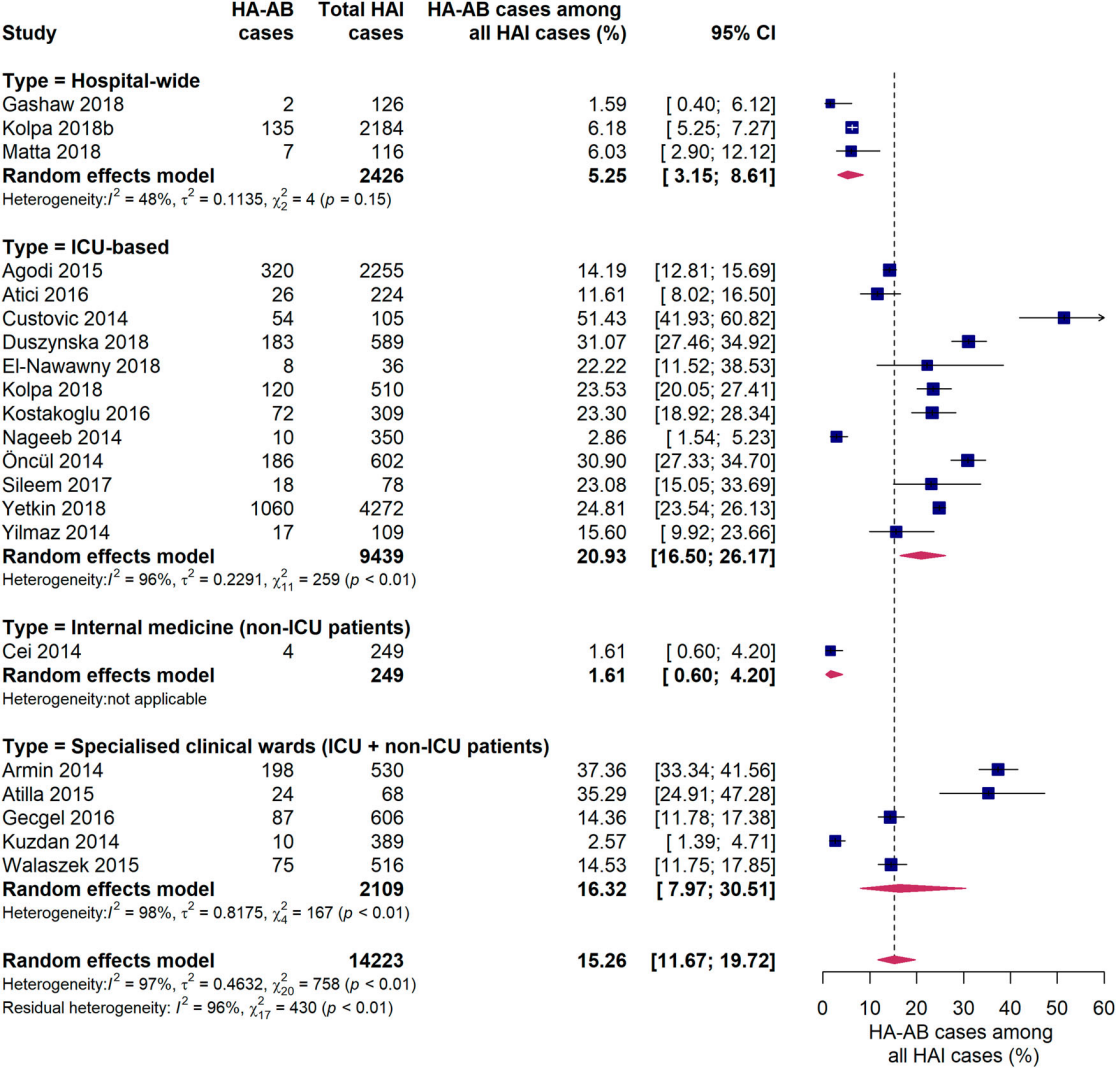
Forest plot of studies on the proportion of hospital-acquired *Acinetobacter baumannii* infections among all hospital-acquired infections. Abbreviations: HAI: hospital-acquired infections, HA-AB: hospital-acquired *Acinetobacter baumannii*, ICU: intensive care unit, 95% CI: 95% confidence interval.

21 incidence studies reported the proportion of *A. baumannii* as a causative agent for hospital-acquired infections. The pooled estimate of the proportion of *A. baumannii* associated infections among all HAIs was 15.3% (95% CI 11.7-19.7%) (Figure 6). Similar to the analyses described above, the burden of *A. baumannii* was especially high in intensive care units, where *A. baumannii* infections accounted for 20.9% (95% CI 16.5-26.2%) of all hospital-acquired infections, although considerable inter-study variation was observed (*I*^2^ = 96%, *p* < 0.01). In studies conducted in specialist clinical departments/wards comprised of ICU and non-ICU patients, the pooled estimate of the proportion of HA-AB infections among all HAIs was 16.3% (95% CI 8.0-30.5%). However, a large variance and inter-study variation (*I*^2^ = 98%, *p* < 0.01) was found. As reported by three studies conducted at hospital-wide level, *A. baumannii* was responsible for 1.6% to 6.2% of all HAIs (pooled estimate: 5.3% [95% CI 3.2-8.6%]). In one study conducted solely on an internal medical ward, *A. baumannii* was identified as the causative pathogen in 1.6% of all HAIs. Although the proportion of HA-AB among all HAIs on ICUs observed in the EUR WHO region was about twice as large as in the EMR WHO region, the difference was not statistically significant (23.6% [95% CI 17.0-31.8%] vs. 12.0% [95% CI 2.8-38.8%], *Q* = 1.03, *p* = 0.310). Furthermore, no significant differences in the proportion of HA-AB among all HAIs in the ICU were found among country income levels (high income [four studies], upper-middle income [six studies] and lower-middle income countries [two studies], *Q* = 1.40, *p* = 0.4972).

**Figure 7.**
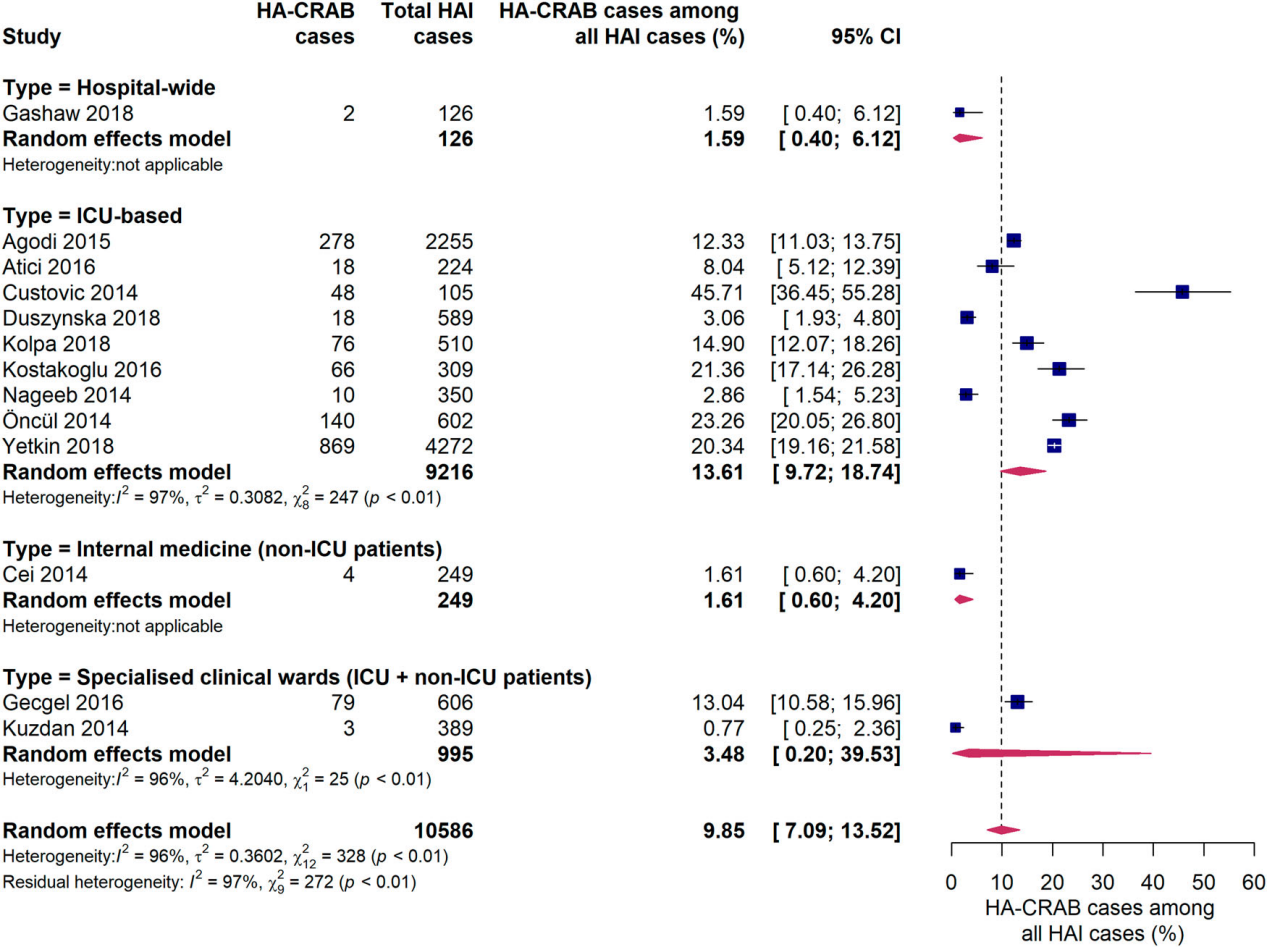
Forest plot of studies on the proportion of hospital-acquired carbapenem-resistant *Acinetobacter baumannii* infections among all hospital-acquired infections. Abbreviations: HAI: hospital-acquired infections, HA-CRAB: hospital-acquired carbapenem-resistant *Acinetobacter baumannii*, ICU: intensive care unit, 95% CI: 95% confidence interval.

Thirteen studies reported data on the proportion of carbapenem-resistant *A. baumannii* infections among all hospital-acquired infections (Figure 7). In ICUs, the pooled proportion of HA-CRAB infections among all HAIs was 13.6% (95% CI 9.7-18.7%). However, a large inter-study variation was observed (*I*^2^ = 97%, *p* < 0.01), with individual estimates ranging from 2.9% to 45.7%. There is evidence that the proportion of HAIs due to CRAB was much lower outside ICUs, as reported in one hospital-wide study and another conducted on an internal medical ward. Both studies found that 1.6% of all HAIs were caused by carbapenem-resistant *A. baumannii*. No comparison of WHO regions was performed since only one study from the AFR WHO region and none from the EMR WHO region were included in this subgroup analysis. Interestingly, in studies conducted in upper-middle income countries (*n* = 5) the proportion of HA-CRAB infections among all HAIs in the ICU was markedly higher compared to studies (*n* = 3) from high income countries (21.9% [95% CI 16.2-28.9%] vs. 8.8% [95% CI 4.8-15.6%], *Q* = 7.77, *p* = 0.0053).

### Prevalence of hospital-acquired *A. baumannii* infections

Two multicenter hospital-wide point prevalence studies were included in this review that reported prevalences of 3.0 and 10.6, respectively, hospital-acquired *A. baumannii* infections per 1,000 patients. Interestingly, while Ahoyo et al. 2014 (Benin) [36] observed no infections with carbapenem-resistant *A. baumannii*, Kritsotakis et al. 2017 (Greece) [40] found that among all hospital-acquired *A. baumannii* infections, 92% were carbapenem-resistant, resulting in a HA-CRAB prevalence of 8.9 per 1,000 patients.

Both studies also provided hospital-wide data on the proportion of *A. baumannii* among all pathogens causing hospital-acquired infections. Kritsotakis et al. 2017 [40] reported that *A. baumannii* was responsible for 10.7% of all HAIs. In contrast, Ahoyo et al. 2017 [36] identified *A. baumannii* as the causative pathogen in only 1.3% of all HAIs.

## Discussion

Hospital-acquired infections caused by *Acinetobacter baumannii*, particularly carbapenem-resistant strains, are a significant threat to health and patient safety worldwide as a result of the continuing decline in treatment options. This study is the first systematic review and meta-analysis to provide a comprehensive summary of recent data on the incidence and prevalence of nosocomial infections with (carbapenem-resistant) *Acinetobacter baumannii* in the WHO-defined regions of Europe, Africa and the Eastern Mediterranean. Based on a review of 24 included studies published between 2014 and 2018, we show that hospital-acquired infections caused by *Acinetobacter baumannii* represent a significant risk to health for hospital patients in Europe, Africa and Eastern Mediterranean regions.

Importantly, the incidence of hospital-acquired AB infections is particularly high in ICUs, where the pooled estimate is as high as 56 HA-AB cases per 1,000 ICU patients and the pooled incidence density is 4.4 HA-AB cases per 1,000 patient ICU days. Due to vulnerable patients with severe comorbidities and high number of invasive procedures, ICUs are known hotbeds for healthcare-acquired infections, including *Acinetobacter* species [49,50]. Our analyses showed that there are no systematic differences in the incidence of hospital-acquired *A. baumannii* infections in ICUs between the European and the Eastern Mediterranean WHO regions. It is important to note that our European study set is over-represented by countries located in the south-eastern part of the European WHO region. However, studies conducted in Italy and Poland found similar ICU incidence when compared to studies in Bosnia–Herzegovina, Turkey and countries from the EMR WHO-region.

In addition to the high incidence of HA-AB infections in ICUs, we found that the pooled incidence of carbapenem-resistant *A. baumannii* infections is 42 HA-CRAB cases per 1,000 ICU patients, with individual study estimates ranging between 7 and 213 cases per 1,000 ICU patients. In comparison, a recent systematic review reported incidences of 18–649 HA-CRAB cases per 1,000 ICU patients in Southeast Asia [51]. Importantly, the pooled HA-CRAB incidence observed in our study is only slightly lower than the total incidence of HA-AB infections, suggesting that the majority of hospital-acquired *A. baumannii* infections on ICUs are caused by carbapenem-resistant strains. This finding is particularly concerning since therapeutic options to treat CRAB infections are limited and these infections are generally associated with high morbidity, mortality and treatment costs [2,3,52].

Not surprisingly, our study provides evidence that the incidence and prevalence of HA-AB infections is 10–50 times lower in hospital-wide studies compared to ICU-based studies. Two studies from Benin [36] and Greece [40] showed prevalence of 3 and 10.6 AB infections per 1,000 patients, respectively. In comparison, large multicenter studies from the United States and China reported prevalences of 0.7 HA-AB cases [53] and 3.2 HA-AB cases [54] per 1,000 patients, respectively. Moreover, evidence from a very recent study also shows that the prevalence of carbapenem-non-susceptible *Acinetobacter* spp. in the United States (0.3 cases per 1000 admissions) is lower than in the WHO-regions analysed in this study [55].

The prominent role of hospital-acquired *A. baumannii* infections in ICUs is underlined by our finding that *A. baumannii* is the causative pathogen in more than 20% of all nosocomial infections. In fact, as shown by several large studies in our study set, *A. baumannii* is the most frequent organism to be responsible for nosocomial infections in the ICU, with higher infection rates than other clinically relevant pathogens, such as *K. pneumoniae*, *E. coli* and *S. aureus* [16,25,34]. This high proportion of *A. baumannii*-associated HAIs on ICUs is also found in other parts of the world, including Southeast Asia [56–58], China [59] and Latin America [60]. In addition, a review that examined studies between 1995 and 2008 from low and middle income countries also found that 19% of all HAIs are caused by *Acinetobacter* spp. in intensive care, and burn and transplant units [61]. In contrast, healthcare-acquired infections with *Acinetobacter* spp. are less prevalent on ICUs in the United States, where only 1.1% of all HAIs on ICUs are caused by *Acinetobacter* spp. [62]. According to 2016 data from The European Surveillance System (TESSy), *Acinetobacter spp.* causes only 2 - 8% of HAIs in ICUs [63] in Europe; profoundly less than our estimates. These discrepancies may be explained by the fact that many Western and Northern European countries provide data on hospital-acquired infections in TESSy, while in our analysis studies from countries in these regions were disproportionately under-represented. TESSy data show that *Acinetobacter* spp. cause more HAIs in ICUs in eastern and southern countries compared to countries in northern and western regions of WHO-Europe. It is interesting that studies providing hospital-wide incidence estimates show that *A. baumannii* causes only 1.6 - 6% of all hospital-acquired infections compared to 20% in the ICU (Figure 2). Other pathogens such as *S. aureu*s and *E. coli* are more frequently found among hospital-acquired infections on a hospital-wide level than *A. baumannii,* as shown by several studies in Europe and Africa [37,38,42]. In comparison to our data, a large multicenter study including 52 hospitals found that 7.9% of all HAIs in China are caused by *A. baumannii* [54]. Interestingly, while our data indicate similar proportions of HA-AB infections among all HAIs between high (22%) and upper-middle (25%) income countries, we observed that HA-CRAB proportions among all HAIs are much lower in high income countries (9%) compared to upper-middle income countries (22%). These findings suggest that carbapenem resistance proportions of *A. baumannii* are much higher in the ICU in countries with upper-middle income emphasizing the clinical relevance of HA-CRAB in the ICU of middle income countries.

There is a general dearth of recent studies investigating the burden of (carbapenem-resistant) *Acinetobacter baumannii* infections in the African WHO region. However, available evidence suggests the widespread prevalence of carbapenemase-producing bacteria in Africa, with prevalence ranging from 2.3% to 67.7% in North Africa, and from 9% to 60% in sub-Saharan Africa [64]. Studies to delineate the magnitude and spread of *A. baumannii* infections across Africa are therefore urgently needed. In addition, the Eastern Mediterranean region is affected by several difficulties that are complicating issues around the spread and control of antimicrobial resistance (AMR) [65]. It is not surprising that the burden of AMR and CRAB in particular remains high in this region, as identified in studies including our own [10].

### Strength and limitations

With 24 included studies and data from more than 280,000 hospitalized patients, our systematic review represents the largest study on the incidence and prevalence of hospital-acquired infections from (carbapenem-resistant) *A. baumannii* in the European, African and Eastern Mediterranean WHO regions. A major strength of our study is that we only included studies comprising of non-disease-specific patients from whole hospitals or individual wards, which ensured the representativeness of our estimates for these institutions and wards. However, our study has some limitations. Firstly, the country representativeness of the individual studies is unclear in most cases, which limits the external validity of our findings. Secondly, studies are not evenly distributed across the WHO regions. In particular, the WHO EUR study set is over-represented by studies from Turkey, while no studies from western and northern parts of Europe were included. Consequently, possible differences in (CR)AB incidence and prevalence between the EUR and EMR WHO regions may be masked by the geographical proximity of Turkey to the Eastern Mediterranean WHO region. Thirdly, due to the relatively low number of hospital-wide studies, our hospital-wide estimates of hospital-acquired *A. baumannii* infections are unlikely to be generalizable. Finally, we observed a large inter-study heterogeneity, particularly within the ICU study set, and the pooled effect sizes must therefore be interpreted with caution. Likely reasons for the large inter-study variations are differences in the distribution of underlying diseases among the included patient cohorts, surgical procedures, immune status and mechanical ventilation. These factors are associated with an increased risk of hospital-acquired infections [66]. Differences in environmental prevalence and colonization rates by *Acinetobacter* spp., both of which are understudied, may also affect HA-AB and HA-CRAB infection rates. In addition, we also recognize that it is difficult to adequately compare outcomes of pathogen-identification tests and antimicrobial susceptibility tests carried out across various regions due to non-standardization of definitions, infection sources and laboratory methods.

### Conclusion

Our study stresses the relevance of hospital-acquired infections caused by *A. baumannii*, including infections with carbapenem-resistant strains, in the European, African and Eastern Mediterranean WHO regions. In particular, the evidence provided in our systematic review highlights the prominent role of *A. baumannii* as an “ICU pathogen”, particularly in light of the high carbapenem-resistant rates observed in most of the included studies. This finding can guide ICUs to prioritize their allocation of resources for infection prevention and control, and in the stewardship of antibiotics, particularly those on the WHO’s Essential Antibiotics Watch and Reserve categories [67].

## Supplementary Material

Supplemental Material

## Geolocation information

Eastern Mediterranean Region, Europe, Africa
