# Supplementary material for: The incidence and prevalence of hospital-acquired (carbapenem-resistant) Acinetobacter baumannii in Europe, Eastern Mediterranean and Africa: a systematic review and meta-analysis
Source: Emerg Microbes Infect. 2019 Dec 5;8(1):1747–59. doi: 10.1080/22221751.2019.1698273 (PMC6913636; doi:10.1080/22221751.2019.1698273)
Supplement: Supplemental Material [file TEMI_A_1698273_SM2890.docx]

**Additional file 1**

**Search string in MEDLINE and EMBASE:**

('acinetobacter baumannii' OR 'a. baumannii' OR 'acinetobacter baumannii spp*' OR 'acinetobacter spp*' OR 'a. baumannii spp*' OR 'acinetobacter complex' OR 'acinetobacter baumannii species' OR 'a. baumannii species') AND (incidence OR prevalence OR 'epidemiological data' OR 'epidemiolog*' OR 'frequenc*' OR population OR 'population-based' OR surveillance OR outbreak OR 'health survey') AND [2014-2019]/py

**Search string in The Global Index Medicus:**

Search string: Acinetobacter OR baumannii

Fields: Title, abstract, subject

Filter: Index Medicus for the Eastern Mediterranean Region (IMEMR), African Index Medicus (AIM)

**Additional file 2**

**Additional Fig.1**

**
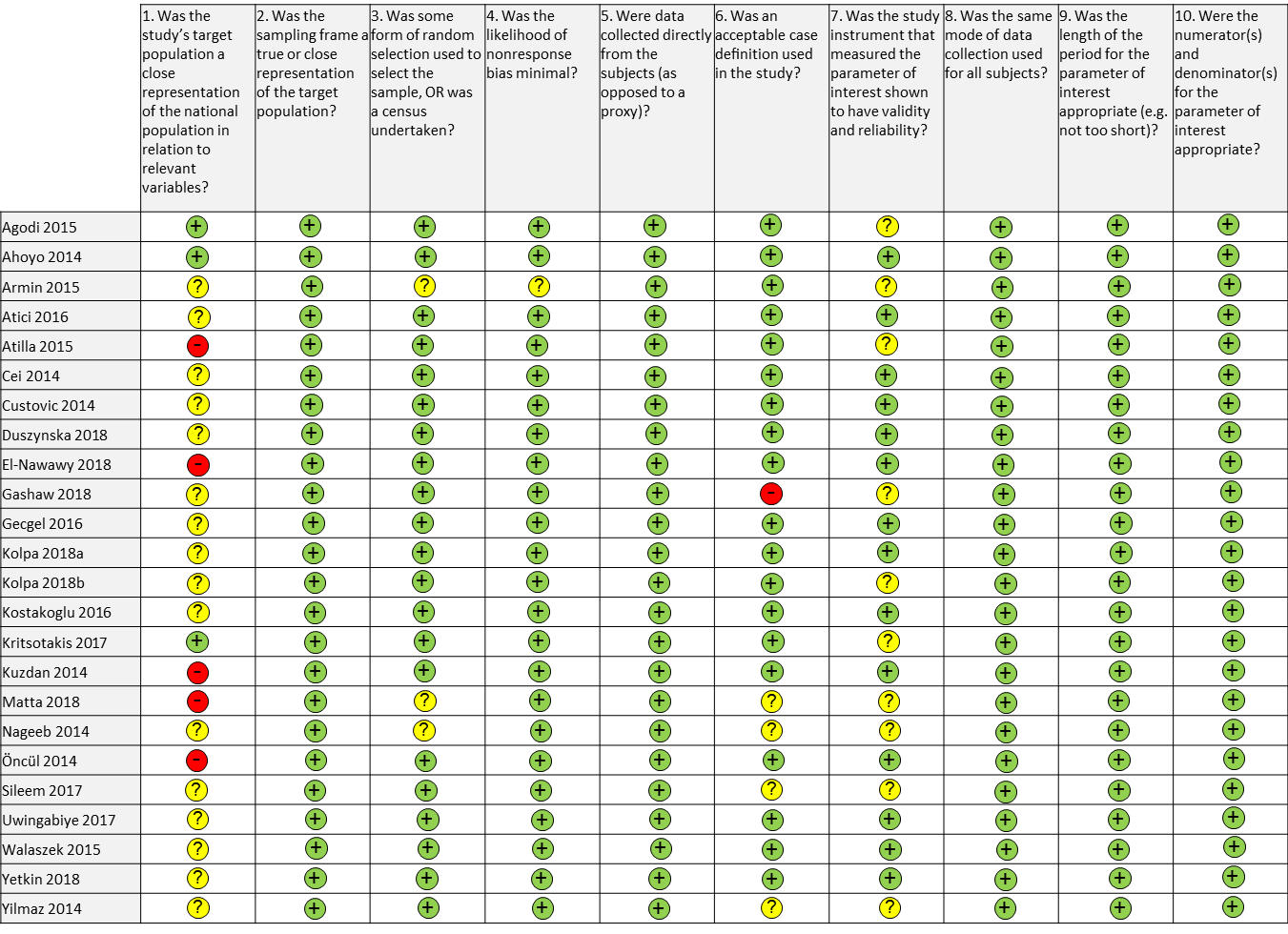
**

**Additional Figure 1.** Risk of bias in the included studies based on Hoy et al. [13]

Green dot: low risk of bias, red dot: high risk of bias, yellow dot: unclear
